# Hypergraph-based contrastive learning for enhanced fraud detection

**DOI:** 10.3389/frai.2025.1703135

**Published:** 2025-11-26

**Authors:** Qinhong Wang, Yiming Shen, Husheng Dong

**Affiliations:** 1 School of Computer Engineering, Suzhou Polytechnic University, Suzhou, China; 2 College of Science Mathematics and Technology, Wenzhou-Kean University, Wenzhou, China

**Keywords:** fraud detection, gated hypergraph convolution, contrastive learning, multi-relational fusion, hyperedge levels

## Abstract

The proliferation of digital platforms has enabled fraudsters to deploy sophisticated camouflage techniques, such as multi-hop collaborative attacks, to evade detection. Traditional Graph Neural Networks (GNNs) often fail to capture these complex high-order patterns due to limitations including homophily assumption failures, severe label imbalance, and noise amplification during deep aggregation. To address these challenges, we propose the Hypergraph-based Contrastive Learning Network (HCLNet), a novel framework integrating three synergistic innovations. Firstly, multi-relational hypergraph fusion encodes heterogeneous associations into hyperedges, explicitly modeling group-wise fraud syndicates beyond pairwise connections. Secondly, a multi-head gated hypergraph aggregation mechanism employs parallel attention heads to capture diverse fraud patterns, dynamically balances original and high-order features via gating, and stabilizes training through residual connections with layer normalization. Thirdly, hierarchical dual-view contrastive learning jointly applies feature masking and topology dropout at both node and hyperedge levels, constructing augmented views to optimize self-supervised discrimination under label scarcity. Extensive experiments on two real-world datasets demonstrate HCLNet's superior performance, achieving significant improvements over the baselines across key evaluation metrics. The model's ability to reveal distinctive separation patterns between fraudulent and benign entities underscores its practical value in combating evolving camouflaged fraud tactics in digital ecosystems.

## 1 Introduction

The rapid proliferation of digital ecosystems has brought about unprecedented convenience while also giving rise to increasingly sophisticated fraud patterns. In domains spanning e-commerce, financial services, and social media, fraudulent activities such as fake reviews, payment scams, and bot-driven spam collectively incur annual losses exceeding $50 billion globally (Shehnepoor et al., 2021; Zou and Cheng, 2025). Fraudsters now frequently employ coordinated strategies such as multi-hop or multi-account camouflage to evade detection in environments including e-commerce, financial transactions, and social platforms. Traditional rule-based systems and unimodal statistical approaches often fall short in identifying such collaborative or cross-modal fraudulent behaviors. Moreover, the shift toward data-driven decision-making has simultaneously introduced a new threat surface: adversarial attacks that deliberately target machine learning models, thereby undermining their reliability and trustworthiness (Lunghi et al., 2023).

Graph Neural Networks (GNNs) have emerged as a dominant paradigm in fraud detection for their ability to model relational and topological structures. By propagating information along edges, GNNs capture local dependencies between entities (e.g., user-device-transaction triads), achieving state-of-the-art results in benchmarks like YelpChi and Amazon (Dou et al., 2020). However, GNN-based detectors face three critical challenges: (1) **Homophily assumption failure**, where the fundamental GNN principle that similar nodes connect is violated in fraud detection because fraudulent entities often camouflage themselves by interacting with legitimate nodes, leading to inaccurate information propagation and reduced detection efficacy; (2) **Extreme label imbalance**, as fraudulent nodes typically constitute less than 5% of graphs (Table 1), causing models to bias toward majority classes (Wang X. et al., 2023), with the scarcity of labeled frauds limiting supervised signal propagation and oversampling techniques often introducing synthetic patterns absent in real attacks (Xie et al., 2023); and (3) **Inadequate high-order modeling** stems from standard graphs' inability to capture the multi-node interactions inherent in fraud rings (e.g., collusive spamming). Specifically, pairwise edges cannot represent n-ary relations, such as a group of users coordinating fake reviews across products. This leads to fragmented detection of syndicate behaviors. Furthermore, existing solutions like meta-paths require manual design and lack adaptability to evolving fraud tactics (Qin et al., 2022). Hypergraphs offer a natural solution but struggle with noise amplification in deep aggregation (Yuan et al., 2022). Additionally, the dynamic evolution of fraud patterns—where tactics continuously adapt to bypass detection systems—demands models capable of online adaptation without catastrophic forgetting.

**Table 1:** Statistics of two datasets.

**Dataset**	**#Nodes (Fraud%)**	**#Features**	**Class**	**$Class**	**Relation**	**#Relations**
YelpChi	45,954 (14.53%)	32	Positive	6,677	R-U-R	49,315
					R-S-R	3,402,743
			Negative	39,277	R-T-R	573,616
Amazon	11,944 (6.87%)	25	Positive	821	U-P-U	175,608
					U-S-U	3,566,479
			Negative	11,123	U-V-U	1,036,737

Despite these advances, existing methods exhibit clear limitations in holistically addressing the triad of challenges: homophily violation, label scarcity, and high-order relational modeling. For instance, while spectral models like BWGNN (Tang et al., 2022) and GHRN (Gao et al., 2023) effectively handle heterophily, they rely on global graph decomposition and scale poorly. Similarly, contrastive learning frameworks such as CONSISGAD (Chen et al., 2024) and POCL (Zhang et al., 2024) mitigate label imbalance but are confined to pairwise graphs, failing to capture group-level fraud semantics. Hypergraph-based approaches like TROPICAL (Haghighi et al., 2025) model high-order relations yet lack explicit contrastive supervision to enhance discriminability under extreme imbalance.

To bridge these gaps, we propose Hypergraph-based Contrastive Learning Network (HCLNet), a novel framework that combines hypergraph structure modeling and self-supervised contrastive learning. Firstly, the multi-relational hypergraph fusion encodes diverse associations into hyperedges to model collaborative fraud rings (Section 3.1), which solves the problem of inadequate high-order modeling by explicitly capturing multi-node interactions beyond pairwise connections. Secondly, the multi-head gated hypergraph aggregation mechanism (Section 3.2) adaptively fuses raw features with hypergraph semantics to suppress noise propagation. This approach addresses homophily assumption failure by employing parallel attention heads for handling heterogeneous node connections and gating mechanisms for balancing feature aggregation. Thirdly, the hierarchical dual-view contrastive learning (Section 3.3) constructs augmented views at both the node and hyperedge levels via self-supervision. This enhances fraud pattern discrimination, alleviates label scarcity, and tackles extreme label imbalance by leveraging unlabeled data through dual-view self-supervised optimization. Our contributions are summarized as follows:

- **A hypergraph-contrastive fusion framework for fraud detection**. We propose a multi-relational hypergraph fusion method that explicitly models high-order fraud patterns through hyperedges, aiming to address the limitations of pairwise connections in capturing collaborative fraud patterns within traditional graph structures.
- **Multi-head gated aggregation with noise suppression**. We design a hypergraph aggregation mechanism with parallel attention heads and dynamic feature balancing gates, which helps mitigate noise propagation while preserving discriminative signals under heterophily conditions, thereby enhancing robustness against camouflaged fraudsters.
- **Hierarchical dual-view contrastive learning under label scarcity**. We develop a self-supervised framework that coordinates node-level and hyperedge-level representations through dual-view augmentation, demonstrating effectiveness in handling label imbalance and achieving competitive performance on real-world benchmarks.

Through extensive experiments, we demonstrate that HCLNet achieves state-of-the-art performance on real-world fraud detection benchmarks, effectively reconciling the limitations of existing approaches while offering interpretable and scalable fraud analysis.

## 2 Related works

### 2.1 Fraud detection

Predominant approaches in fraud detection fall into two primary categories: homophily-enhancing models that strengthen connections between similar nodes through edge re-weighting (Dou et al., 2020), although manual graph modifications may introduce bias. The second category includes spectral adaptive models that utilize band-pass filters to address heterophily (e.g., Beta wavelets in BWGNN Tang et al., 2022 or high-pass filters in GHRN Gao et al., 2023), but their dependence on global graph spectrum decomposition hinders scalability. In fraudulent review detection—particularly for platforms like Amazon and Yelp—recent studies highlight three persistent challenges: extreme label imbalance where fraudulent reviews constitute < 5% of datasets (Shehnepoor et al., 2021), bot-generated content that mimics genuine patterns (Yao et al., 2017), and cold-start scenarios for new users/items (Wang et al., 2017). To mitigate label imbalance, researchers have developed multi-modal frameworks: Luca and Zervas (2016) integrates behavioral patterns (e.g., rating bursts and review gaps) to amplify sparse fraud signals; (Li et al., 2016) focuses on textual features (e.g., linguistic anomalies and sentiment inconsistencies) to identify subtle fraudulent cues; and Shehnepoor et al. (2017) utilizes graph structures to propagate limited labels through relational contexts. For bot-generated content, GAN-based approaches (Aghakhani et al., 2018) generate synthetic fraud patterns to enhance detector robustness. To address cold-start issues, attribute-enhanced domain adaptation (You et al., 2018) leverages cross-domain feature transfer. Despite these advances, most systems remain challenged by coordinated group frauds due to their dependence on pairwise structures. Recent work like POCL (Zhang et al., 2024) addresses temporal dynamics through contrastive learning but remains confined to pairwise graphs, limiting its ability to capture multi-node fraud rings.

A recent line of research specifically targets the challenge of learning under extremely limited labels. SpaceGNN (Dong et al., 2025) projects nodes into multiple latent spaces and employs distance-aware propagation to enhance information aggregation, effectively identifying anomalies when labeled data is scarce. Similarly, CGNN (Li et al., 2025) enriches node representations by constructing a context graph that captures long-range dependencies and high-order interactions, demonstrating strong performance in fraud detection with minimal supervision. Another notable approach, LGM-GNN (Li et al., 2023), introduces a memory module to record and integrate both local neighborhood patterns and global graph-level prototypes, thereby strengthening the model's ability to discern fraudulent patterns from a holistic perspective. Despite their effectiveness in handling label scarcity, these methods—including the previously discussed CONSISGAD and POCL are inherently built upon pairwise graph structures. This fundamental limitation restricts their capacity to explicitly model the complex, multi-node collaborative relationships that are characteristic of organized fraud rings.

### 2.2 Hypergraph

Hypergraphs, where each hyperedge connects multiple nodes, provide a natural mechanism for representing high-order relationships that are common in fraud rings, coordinated attacks, or group-based manipulations. Unlike traditional graphs, which are limited to binary relations, hypergraphs can encode multi-party transactions or multi-hop behaviors in a single structure. Recent advancements in hypergraph representation learning have demonstrated the effectiveness of capturing high-order interactions in various domains. For example, HyperGCN (Yadati et al., 2019) extends Graph Convolutional Networks (GCNs) to hypergraphs by aggregating information across hyperedges, enabling the modeling of complex group-wise relationships. Similarly, HyperPath-based representation learning (Huang et al., 2019) introduces a framework for hyper-networks by leveraging hyper-paths to capture sequential dependencies, which could be adapted for detecting evolving fraud patterns. UniGNN (Huang and Yang, 2021) proposes a unified framework for both graph and hypergraph neural networks, emphasizing the importance of hypergraph structures in tasks requiring high-order relational modeling. These approaches highlight the potential of hypergraphs in encoding collaborative fraud behaviors that traditional pairwise graphs cannot capture. Several works have explored hypergraph-based methods for recommendation systems and community detection, but their use in fraud detection remains limited. Models like GAGA (Wang Y. et al., 2023) attempt to Mitigating low homophily via neighborhood label aggregation but fail to utilize contrastive supervision or exploit the full semantic diversity of real-world interactions. Our method advances this line of work by fusing multi-relational edge types into hyperedges and learning discriminative embeddings through multi-head gated attention.

### 2.3 Contrastive learning

In the field of fraud detection, contrastive learning has gained significant attention for its ability to address core challenges including label scarcity, data heterogeneity, and complex relationship modeling. Traditional GNN-based fraud detection methods are often constrained by the homophily assumption. However, fraudulent entities frequently camouflage themselves by interacting with benign nodes, leading to distorted information propagation. Contrastive learning leverages unlabeled data through self-supervised mechanisms to enhance representation discriminability: for instance, the instance discrimination task treats each instance as an independent class, generates positive samples, and obtains negative samples through a global memory bank and Noise Contrastive Estimation (NCE) (Wu et al., 2018). This drives the model to learn distinctive features of fraudulent entities. Nevertheless, negative samples in fraud scenarios may contain semantically similar nodes (e.g., camouflaged fraudsters masquerading as legitimate users). Directly repelling such samples can degrade model performance. To mitigate this, clustering-enhanced contrastive methods (Li et al., 2020; Caron et al., 2020) introduce the prototype contrastive loss (ProtoNCE), which employs cluster centers as proxy negative samples to alleviate erroneous repulsion. For heterogeneous graphs, recent structure-enhanced methods like STENCIL (Zhu et al., 2022) optimize metapath-induced views by mining hard negatives via structural embeddings, synthesizing challenging samples to improve discrimination. Meanwhile, NNCLR (Dwibedi et al., 2021) significantly advances self-supervised visual representation learning by utilizing nearest neighbors from a support set as positive examples within a contrastive loss framework. Recently emerged negative-sample-free contrastive learning (Chen and He, 2021; Grill et al., 2020) offers new insights for fraud detection. These methods eliminate explicit negative samples and avoid feature collapse via momentum encoders, prediction heads, and gradient stopping, significantly reducing dependence on batch size. This makes them more adaptable to the extreme imbalance of fraud data. However, existing methods exhibit three key limitations: (1) negative sampling strategies struggle to distinguish semantically similar camouflaged fraudsters, (2) pairwise graph constraints fail to capture multi-node collaborative fraud patterns, and (3) single-level contrastive frameworks lack explicit modeling of group-wise interactions.

## 3 Methodology

**Figure 1:**
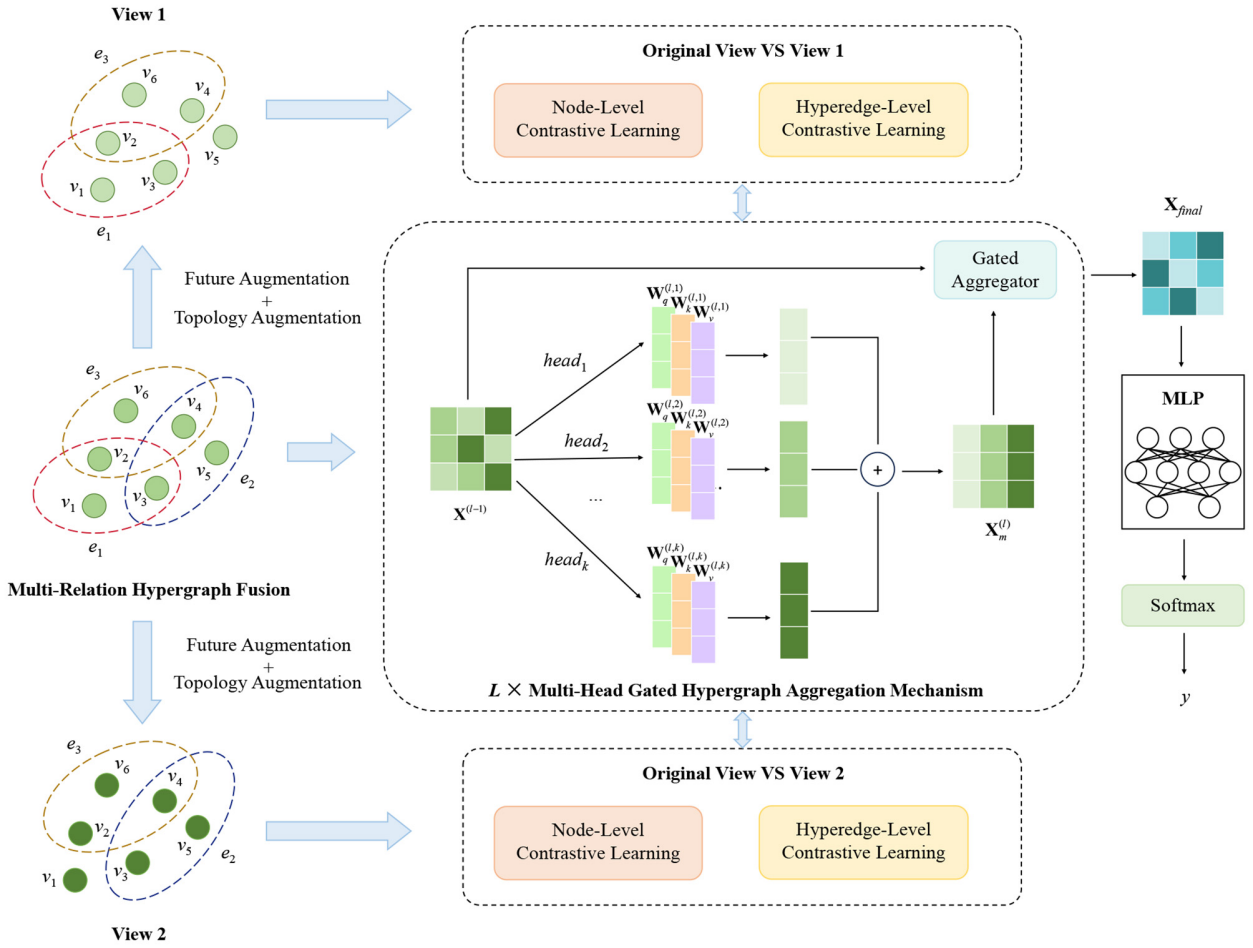
The overview of the proposed HCLNet model.

This section gives the design details of HCLNet, a fraud detection model based on hypergraph and contrast learning, which mainly consists of (1) **Multi-relational hypergraph fusion**, (2) **Multi-head gated hypergraph aggregation mechanism**, (3) **Hierarchical dual-view contrastive learning framework**, and (4) **Prediction and training**. The structure of the model is shown in Figure 1.

### 3.1 Multi-relation hypergraph fusion

For fraud detection tasks, homogeneous nodes such as transaction records and user accounts often form complex networks through multiple types of associations. Traditional GNNs, however, only model pairwise node interactions, struggling to capture multi-node collaborative fraud patterns—such as gang-related collective fraud behaviors. To address this limitation, we design a multi-relation hypergraph using hypergraph theory to uniformly represent heterogeneous interactions among homogeneous nodes and mine high-order collaborative fraud features. Specifically, we define the hypergraph as G=(V,E), where $V=\left\{v_{1},v_{2},\ldots ,v_{n}\right\}$ denotes the set of homogeneous nodes (i.e., n=|V|) and $E=\left\{e_{1},e_{2},\ldots ,e_{m}\right\}$ represents the set of hyperedges (i.e., m=|E|).

The hypergraph construction involves three key steps. Firstly, we symmetrize each original relation r∈R (dataset's relation set) to eliminate directional biases, aligning them with the “reciprocal association” characteristic of fraud behaviors. Next, using the symmetrized relations, we apply the connected component algorithm to extract hyperedge sets $E_{r}$, effectively capturing multi-node collaborative patterns that traditional graph models fail to represent. Finally, we fuse all $E_{r}$ to obtain the global hyperedge set $E={⋃}_{r\in R}E_{r}$ and build a node-hyperedge incidence matrix **H** ∈ {0, 1}^*n*×*m*^. This matrix preserves semantic differences of various relations while establishing cross-relation topological associations via high-order hyperedge connections.

### 3.2 Multi-head gated hypergraph aggregation mechanism

To address the problem that traditional GNNs cannot capture multi-node collaborative fraud patterns, we design a Multi-Head Gated Hypergraph Aggregation Mechanism (MG-HAM). Built upon hypergraphs, MG-HAM first captures fraud patterns in multiple semantic spaces in parallel via multi-head hypergraph convolution. It then uses a dynamic gated aggregator to adaptively balance old and new features, and employs a gated residual connection to ensure stable feature transmission and alleviate gradient issues. Ultimately, it outputs node representations that fuse high-order collaborative patterns with original feature information, thereby significantly improving the accuracy of fraud detection.

#### 3.2.1 Multi-head hypergraph convolution

Multi-head hypergraph convolution combines the multi-head attention mechanism with traditional hypergraph convolution, enabling the multi-dimensional parallel capture of complex fraud patterns. Through multiple independent attention heads, it can simultaneously focus on different types of fraud signals—such as collaborative fraud behaviors and abnormal transaction patterns. Each attention head learns feature representations in a distinct semantic space, thereby enhancing the model's expressive capability. The module first maps node features to the hyperedge space via node-hyperedge aggregation, as shown in Equation 1:


$$\begin{array}{l} {\text{E}}^{\left(l\right)}={\text{H}}^{⊤}{\text{X}}^{\left(l-1\right)} \end{array}$$ (1)

where **H** ∈ {0, 1}^*n*×*m*^ is the incidence matrix, **X**^(*l* − 1)^ ∈ ℝ^*n*×*d*^ is the node feature matrix from the (*l* − 1)-th layer, and *d* is the dimension of node features. **E**^(*l*)^ ∈ ℝ^*m*×*d*^ denotes the hyperedge feature for the *l*-th layer—specifically, it is the average of all node features contained in the corresponding hyperedge.

Subsequently, via *K* independent attention heads, we learn different representation spaces tailored to capture diverse fraud patterns. For the *l*-th layer, this process yields two key outputs: the similarity matrix ${\text{A}}_{m}^{\left(l,k\right)}\in {\mathbb{R}}^{m\times m}$ and the attention-weighted hyperedge feature matrix ${\text{E}}_{m}^{\left(l,k\right)}\in {\mathbb{R}}^{m\times d_{k}}$, as shown in Equations 2, 3:


$$\begin{array}{l} {\text{A}}_{m}^{\left(l,k\right)}=softmax\left(\frac{\left({\text{E}}^{\left(l\right)}{\text{W}}_{q}^{\left(l,k\right)}\right){\left({\text{E}}^{\left(l\right)}{\text{W}}_{k}^{\left(l,k\right)}\right)}^{⊤}}{\sqrt{d_{k}}}\right) \end{array}$$ (2)

$$\begin{array}{l} {\text{E}}_{m}^{\left(l,k\right)}={\text{A}}_{m}^{\left(l,k\right)}\left({\text{E}}^{\left(l\right)}{\text{W}}_{v}^{\left(l,k\right)}\right) \end{array}$$ (3)

where *d*_*k*_ = *d*/*K* stands for the feature dimension of the *k*-th head, ${\text{W}}_{q}^{\left(l,k\right)},{\text{W}}_{k}^{\left(l,k\right)},\text{and}{\text{W}}_{v}^{\left(l,k\right)}\in {\mathbb{R}}^{d\times d_{k}}$ are all learnable parameters.

Then, the enhanced hyperedge features are propagated back to the relevant nodes, completing the closed-loop information transmission of the convolution operation. Finally, the outputs of the *K* attention heads are concatenated, and multi-dimensional fraud features are fused via a linear transformation, as shown in Equations 4, 5:


$$\begin{array}{l} {\text{X}}^{\left(l,k\right)}=\text{H}{\text{E}}_{m}^{\left(l,k\right)} \end{array}$$ (4)

$$\begin{array}{l} {\text{X}}_{m}^{\left(l\right)}=\left[\begin{array}{c} {\text{X}}^{\left(l,1\right)};{\text{X}}^{\left(l,2\right)};\cdots \;;{\text{X}}^{\left(l,K\right)} \end{array}\right]{\text{W}}_{o}^{\left(l\right)} \end{array}$$ (5)

where [;] denotes concatenation, ${\text{X}}^{\left(l,k\right)}\in {\mathbb{R}}^{n\times d_{k}}$ represents the output of the *k*-th head in the *l*-th layer. ${\text{W}}_{o}^{\left(l\right)}\in {\mathbb{R}}^{d\times d}$ is a learnable linear transformation weight matrix that maps the concatenated multi-head features to a unified node feature space, fusing various fraud pattern information captured by different attention heads. ${\text{X}}_{m}^{\left(l\right)}\in {\mathbb{R}}^{n\times d}$ is thus the final output of the *l*-th layer after undergoing multi-head hypergraph convolution.

#### 3.2.2 Dynamic gated aggregator

The multi-head hypergraph convolution extracts high-order collaborative fraud features ${\text{X}}_{m}^{\left(l\right)}$, but the original node features still contain basic information for fraud detection. Therefore, a dynamic gated aggregator is designed, which learns the importance weights of different attention heads through a gating mechanism, highlighting the feature heads that contribute the most to fraud detection and suppressing noise signals. Gate values are calculated based on the original features and determine the fusion ratio between the original features and the high-order features. The process is detailed in Equations 6, 7:


$$\begin{array}{l} {\text{G}}^{\left(l\right)}=\sigma \left({\text{W}}_{g}^{\left(l\right)}{\text{X}}^{\left(l-1\right)}+{\text{b}}_{g}^{\left(l\right)}\right) \end{array}$$ (6)

$$\begin{array}{l} {\text{X}}_{f}^{\left(l\right)}={\text{G}}^{\left(l\right)}⊙{\text{X}}_{m}^{\left(l\right)}+\left(1-{\text{G}}^{\left(l\right)}\right)⊙{\text{X}}^{\left(l-1\right)} \end{array}$$ (7)

where $W_{g}^{\left(l)\right)}\in {\mathbb{R}}^{d\times d},b_{g}^{\left(l\right)}\in {\mathbb{R}}^{d}$ are learnable parameters, σ(·) denotes the sigmoid activation function, and **G**^(*l*)^ ∈ [0, 1]^*n*×*d*^ is a learnable feature-wise gating matrix that balances the ratio of original features to high-order features in an element-wise manner. ${\text{X}}_{f}^{\left(l\right)}\in {\mathbb{R}}^{n\times d}$ is the fused node feature obtained by dynamic gated aggregation of the *l*-th layer.

#### 3.2.3 Gated residual connection

The fused features ${\text{X}}_{f}^{\left(l\right)}$ from the dynamic gated aggregator may exhibit fluctuations in feature distribution, leading to unstable model training. To address this issue, we further propose a gated residual connection, which introduces layer normalization to mitigate these fluctuations while preserving the adaptive properties of the gating mechanism and mitigating the vanishing gradient problem in deep networks. This process is specifically detailed in Equation 8:


$$\begin{array}{l} {\text{X}}^{\left(l\right)}=\text{LayerNorm}\left({\text{X}}_{f}^{\left(l\right)}\right) \end{array}$$ (8)

where LayerNorm(·) denotes the layer normalization, **X**^(*l*)^ ∈ ℝ^*n*×*d*^ represents the node feature matrix updated from the (*l* − 1)-th layer via the MG-HAM. This provides high-quality inputs for subsequent training tasks, not only improving the model's capability to identify fraud patterns, but also enhancing its generalization performance.

For the convenience of subsequent derivation, we abstract the entire mechanism as the function shown in Equation 9:


$$\begin{array}{l} {\text{X}}^{\left(l\right)}={\text{MG-HAM}}^{\left(l\right)}\left({\text{X}}^{\left(l-1\right)},\text{H}\right) \end{array}$$ (9)

### 3.3 Hierarchical dual-view contrastive learning framework

Building upon the MG-HAM, we propose a Hierarchical Dual-View Contrastive Learning Framework (HDV-CL) to further enhance the model's ability to identify fraud patterns and its generalization performance. HDV-CL jointly optimizes node-level and hyperedge-level representation learning while leveraging a dual-view augmentation strategy to address the key challenges of label scarcity and pattern complexity in fraud detection.

#### 3.3.1 Data augmentation

The performance of contrastive learning hinges on high-quality view generation. Considering the characteristics of hypergraph structures and fraud patterns, we propose two complementary data augmentation strategies—feature augmentation and topology augmentation. Feature augmentation generates two independent augmented views by introducing random masking and Gaussian noise to the original node features **X**, as shown in Equation 10:


$$\begin{array}{l} {\text{X}}^{\left(t\right)}=\text{X}⊙{\text{M}}^{\left(t\right)}+\epsilon^{\left(t\right)},\;{\text{M}}^{\left(t\right)}\sim Bernoulli\left(1-\varepsilon \right),\;\epsilon^{\left(t\right)}\sim N\left(0,\omega {\text{I}}^{2}\right) \end{array}$$ (10)

where *t* = 1, 2 denotes the *t*-th augmented view, **M**^(*t*)^ ∈ {0, 1}^*n*×*d*^ is a masking matrix matching the dimension of the embedding vectors. Each element is independently sampled from a Bernoulli distribution, with a 1 − ε probability of being set to zero. ϵ^(*t*)^ denotes Gaussian noise, whose intensity is regulated by ω. This operation simulates feature missing scenarios, compelling the model to focus on critical fraud features.

Topology augmentation randomly drops hyperedges from the incidence matrix **H**, similarly generating two independent augmented views, as shown in Equation 11:


$$\begin{array}{l} {\text{H}}^{\left(t\right)}=\text{H}\left[:,S^{\left(t\right)}\right],\;S^{\left(t\right)}\sim \text{Bernoulli}\left(1-p_{edge}\right) \end{array}$$ (11)

where *S*^(*t*)^ ∈ {0, 1}^*m*^ is a binary selection vector whose dimension matches the number of hyperedges, and *p*_*edge*_ denotes the hyperedge dropout rate. This operation enhances the model's sensitivity to abnormal connection patterns by disrupting local topological structures.

#### 3.3.2 Node-level contrastive learning

Node-level contrastive learning aims to distinguish the semantic differences between benign and fraudulent nodes in the feature space. For the (*l* − 1)-th layer node features **X**^(*l* − 1, *t*)^ after feature augmentation, we use MG-HAM to obtain its high-level representation **X**^(*l, t*)^, as shown in Equation 12:


$$\begin{array}{l} {\text{X}}^{\left(l,t\right)}={\text{MG-HAM}}^{\left(l\right)}\left({\text{X}}^{\left(l-1,t\right)},{\text{H}}^{\left(t\right)}\right) \end{array}$$ (12)

Subsequently, for the node representations *v*_*i*_ from **X**^(*l*)^ and $v_{i}^{\left(l,t\right)}$ from **X**^(*l, t*)^, we map them to the contrastive space via the same MLP projection head *g*_ϕ_(·), followed by L2 normalization, as shown in Equation 13:


$$\begin{array}{l} z_{i}^{\left(l\right)}=\frac{g_{\phi }\left(v_{i}^{\left(l\right)}\right)}{∥g_{\phi }\left(v_{i}^{\left(l\right)}\right){∥}_{2}},\;z_{i}^{\left(l,t\right)}=\frac{g_{\phi }\left(v_{i}^{\left(l,t\right)}\right)}{∥g_{\phi }\left(v_{i}^{\left(l,t\right)}\right){∥}_{2}} \end{array}$$ (13)

Then, for the *l*-th layer, we employ the InfoNCE loss function to compute the node-level contrastive loss between the original view and the *t*-th augmented view, as shown in Equation 14:


$$\begin{array}{l} L_{node}^{\left(l,t\right)}=-\frac{1}{N}\overset{N}{\underset{i=1}{\sum }}\log\frac{\exp\left(z_{i}^{\left(l\right)⊤}z_{i}^{\left(l,t\right)}/\tau \right)}{\sum_{j=1}^{N}\exp\left(z_{i}^{\left(l\right)⊤}z_{j}^{\left(l,t\right)}/\tau \right)} \end{array}$$ (14)

where τ is the temperature hyperparameter to scale the similarity distribution, and *N* denotes the number of negative samples.

#### 3.3.3 Hyperedge-level contrastive learning

Hyperedge-level contrastive learning aims to enhance the model's ability to perceive high-order semantic structures. To begin with, as per Equation 1, we can compute the representations **E**^(*l, t*)^ of **E**^(*l*)^ across the two augmented views. For the hyperedge representations $e_{c}^{\left(l\right)}$ from **E**^(*l*)^ and $e_{c}^{\left(l,t\right)}$ from **E**^(*l, t*)^, we use a MLP projection head *g*_ψ_(·) different from the one in node-level contrastive learning to map them to the contrastive space and perform normalization, as shown in Equation 15:


$$\begin{array}{l} z_{c}^{\left(l\right)}=\frac{g_{\psi }\left(e_{c}^{\left(l\right)}\right)}{∥g_{\psi }\left(e_{c}^{\left(l\right)}\right){∥}_{2}},\;z_{c}^{\left(l,t\right)}=\frac{g_{\psi }\left(e_{c}^{\left(l,t\right)}\right)}{∥g_{\psi }\left(e_{c}^{\left(l,t\right)}\right){∥}_{2}} \end{array}$$ (15)

Then, for the *l*-th layer, we employ the InfoNCE loss function to compute the hyperedge-level contrastive loss between the original view and the *t*-th augmented view, as shown in Equation 16:


$$\begin{array}{l} L_{hyper}^{\left(l,t\right)}=-\frac{1}{M}\overset{M}{\underset{c=1}{\sum }}\log\frac{\exp\left(z_{c}^{\left(l\right)⊤}z_{c}^{\left(l,t\right)}/\tau \right)}{\sum_{d=1}^{M}\exp\left(z_{c}^{\left(l\right)⊤}z_{d}^{\left(l,t\right)}/\tau \right)} \end{array}$$ (16)

where *M* denotes the number of negative hyperedges.

#### 3.3.4 Hierarchical dual-view contrastive integration

Finally, by hierarchically integrating node-level and hyperedge-level contrastive learning from dual views and fusing multi-layer representations, we define the total contrastive loss as the average of the contrastive losses from the two pairs of views, as shown in Equation 17:


$$\begin{array}{l} L_{cl}=\frac{1}{2}\overset{2}{\underset{t=1}{\sum }}\left[\frac{1}{L}\overset{L}{\underset{l=1}{\sum }}\left(L_{node}^{\left(l,t\right)}+L_{hyper}^{\left(l,t\right)}\right)\right] \end{array}$$ (17)

where *L* denotes the total number of layers in the network. Through this HDV-CL, the model can leverage self-supervised contrastive loss to enhance its representation capability when labeled data is scarce, thereby improving the accuracy of fraud classification tasks.

### 3.4 Prediction and training

HCLNet takes the output **X**_*final*_ from the final layer of MG-HAM and feeds it into a two-layer MLP for prediction, as shown in Equation 18:


$$\begin{array}{l} \hat{\text{Y}}=\text{softmax}\left({\text{W}}_{2}\cdot \text{ReLU}\left({\text{W}}_{1}\cdot {\text{X}}_{final}\right)\right) \end{array}$$ (18)

where $\hat{\text{Y}}$ contains the class probability distribution vector for each node. The model uses this output to compute the class-weighted cross-entropy loss $L_{cls}$, which combines with the hierarchical contrastive loss $L_{cl}$ to form the total loss function, as defined in Equations 19, 20:


$${ℒ}_{cls}=-\frac{1}{n}\sum_{i=1}^{n}w_{y_{i}}y_{i}\log({\hat{y}}_{i})$$ (19)

$${ℒ}_{total}={ℒ}_{cls}+\alpha {ℒ}_{cl}$$ (20)

where *y*_*i*_ is the true label of node *v*_*i*_, ŷ_*i*_ is the predicted probability value for the corresponding class in its probability vector, and *n* is the total number of nodes. The class weight *w*_*y*_*i*__ addresses sample imbalance issues, while α serves as an adjustable weighting coefficient for the contrastive loss.

## 4 Experiments

### 4.1 Experimental setup

#### 4.1.1 Dataset and evaluation metrics

We evaluate the proposed HCLNet and all baselines on following two real-world public fraud detection datasets:

- **YelpChi graph dataset** (Rayana and Akoglu, 2015) contains hotel and restaurant reviews collected from the Yelp platform, featuring three relation types: R-U-R (reviews posted by the same user), R-S-R (reviews with identical star ratings for the same product), and R-T-R (reviews posted in the same month for the same product).
- **Amazon graph dataset** (McAuley and Leskovec, 2013) contains musical instrument reviews with three defined relations: U-P-U (users reviewing at least one common product), U-S-U (users having at least one same star rating within 1 week), and U-V-U (users with top-5% mutual review TF-IDF similarities).

During the data preprocessing stage, we extract node features and three heterogeneous relation matrices from both datasets. When the dimensionality of node features exceeds 100, we employ PCA to reduce them to 100 dimensions, preserving the essential feature information. For multi-relational edge processing, we ensure the symmetry of all relation matrices and extract connected components from each relation matrix as hyperedges, retaining only those containing multiple nodes. Subsequently, we merge hyperedges from all relations to construct a hypergraph incidence matrix and apply symmetric normalization, thereby effectively capturing high-order interaction patterns in the graph. For each dataset, following the setup in Tang et al. (2022), Zou and Cheng (2025), and Haghighi et al. (2025), we chronologically select the first 40% as the training set, the middle 40% as the validation set for hyperparameter tuning, and the last 20% as the test set. Table 1 presents detailed statistics of both datasets.

To address class imbalance, we adopt fairness-aware evaluation metrics to avoid bias toward any category (Luque et al., 2019). Referring to relevant studies (Zou and Cheng, 2025; Xie et al., 2023; Wang X. et al., 2023), we comprehensively assess model performance using three metrics, including macro average F1-macro score (F1-macro), the area under the ROC curve (AUC), and geometric mean (GMean). GMean is defined as $\sqrt{\text{sensitivity}\times \text{specificity}}$, effectively measuring model performance on imbalanced data.

#### 4.1.2 Baselines

To evaluate the performance of the proposed model HCLNet, we choose following competitive methods as baselines:

- **GCN** (Kipf, 2016) as a fundamental graph deep learning model integrates node features and graph structural information through local neighborhood aggregation, effectively identifying collaborative fraud patterns, but its transductive learning mechanism limits handling of new nodes.
- **GraphSAGE** (Hamilton et al., 2017) is an inductive graph model that generalizes node features from local neighbors via neighbor sampling and learnable aggregation functions. It is suitable for dynamic fraud scenarios like real-time fake reviews in e-commerce.
- **GPRGNN** (Chien et al., 2020) improves upon GCN's aggregation by introducing learnable personalized PageRank coefficients for weighted multi-hop neighbor combination, capturing long-range dependencies in complex fraud structures.
- **CARE-GNN** (Dou et al., 2020) dynamically selects attribute-similar neighbors through reinforcement learning, constructing an attribute-relation-topology tri-view framework that effectively filters noisy connections and enhances detection robustness.
- **PC-GNN** (Liu et al., 2021) employs confidence propagation to handle label or structural noise, computing initial confidence from node features and iteratively updating it among neighbors, significantly improving fraud node identification accuracy.
- **CONSISGAD** (Chen et al., 2024) employs a learnable data augmentation mechanism to generate augmented samples that preserve label consistency, coupled with a homophily-aware GNN backbone, significantly improving graph anomaly detection under limited supervision.
- **SpaceGNN** (Dong et al., 2025) employs a multi-space graph neural network for node anomaly detection, leveraging learnable space projection and distance-aware propagation to enhance information aggregation and improve detection accuracy under extremely limited labels.
- **GHRN** (Gao et al., 2023) identifies and prunes heterophilic edges using high-frequency signals from the graph spectrum, enhancing anomaly detection.
- **BWGNN** (Tang et al., 2022) employs Beta wavelets to construct spectral-localized band-pass filters that address the 'right-shift' phenomenon in graph spectra, improving anomaly detection.
- **TROPICAL** (Haghighi et al., 2025) introduces a transformer-based hypergraph framework to capture high-order relationships for detecting camouflaged fraudsters, effectively identifying malicious actors blending with normal entities.

#### 4.1.3 Implementation details

For all baselines, if the original hyperparameters are provided, we use them directly; otherwise, the hyperparameter search space is set as follows: learning rate in {0.01, 0.05, 0.001}, dropout in {0.3, 0.4, 0.5, 0.6}, weight decay in {10^-3^, 10^-4^, 10^-5^}, hidden dimension in {16, 32, 64}. For HCLNet, we set dropout to 0.2, learning rate to 0.005, weight decay to 10^-4^, hidden dimension and contrastive projection dimension to 64, contrastive temperature τ to 0.8, and contrastive loss weight α to 0.8. For the MG-HAM, 4 heads (*K*=4) and 4 layers (*L*=4) are used for YelpChi, while 2 heads (*K*=2) and 3 layers (*L*=3) are used for Amazon. For fair comparison, the maximum number of training epochs is set to 500 for all models, optimized using the Adam optimizer. Our implementation is based on PyTorch 2.3.0 (with CUDA 12.1 and Python 3.12), trained on a server equipped with one 48GB NVIDIA Virtual GPU.

### 4.2 Fraud detection performance

Table 2 presents the comparison results of HCLNet with other baselines, where the following observations are noted:

(1) Among baselines, the foundational GCN suffers from limitations of homophily assumption and shallow propagation, yielding the lowest performance. GraphSAGE optimizes the aggregation process through neighbor sampling and excels in capturing local patterns, particularly standing out on the Amazon dataset. However, traditional GNN models generally struggle to effectively model the complex high-order relationships in fraud detection. Additionally, GPRGNN introduces learnable generalized PageRank coefficients to capture long-range fraudulent associations, but it is sensitive to noise and exhibits performance fluctuations.
(2) Among models specifically designed for fraud detection, CARE-GNN demonstrates certain robustness in noisy scenarios through its tri-view framework and reinforcement learning-based neighbor selection. PC-GNN's belief propagation mechanism shows effectiveness in handling label noise. CONSISGAD employs learnable data augmentation but exhibits performance instability with high variance across datasets, while SpaceGNN maintains modest competitiveness through multi-space projection. These methods each have their distinctive features, yet they still face limitations in modeling complex relationships.
(3) Among the spectral analysis-based models, GHRN and BWGNN demonstrate excellent performance on Amazon, with BWGNN emerging as the optimal baseline model in both F1-macro and GMean metrics. This success stems from the Amazon's structural properties, where its relatively balanced node distribution and well-defined heterophilic edge patterns create favorable conditions for spectral methods to effectively identify anomalous high-frequency signals. The smaller node count also contributes to lower computational complexity in these spectral-based approaches.
(4) The recently proposed TROPICAL, which employs a Transformer-based hypergraph learning approach to capture high-order relations, has delivered outstanding performance on YelpChi, establishing itself as the optimal baseline and demonstrating the advantage of hypergraph learning in modeling complex high-order dependencies. Our proposed HCLNet achieves the best overall performance while exhibiting distinct characteristics across datasets. On the large-scale and complex YelpChi, HCLNet improves F1-macro, AUC, and GMean by 2.09%, 2.23%, and 4.03%, respectively, over the best baseline TROPICAL, highlighting its strength in challenging scenarios. Although HCLNet's F1-macro is slightly lower than that of BWGNN and TROPICAL on Amazon, it still leads on the other two metrics. This demonstrates that through MG-HAM, and HDV-CL, HCLNet is able to maintain stable and high performance across diverse fraud detection settings.

**Table 2:** Performance comparison of HCLNet with baselines over two datasets (mean ± standard deviation over ten runs).

**Model**	**YelpChi**			**Amazon**		
	**F1-macro**	**AUC**	**GMean**	**F1-macro**	**AUC**	**GMean**
GCN	0.5661 ± 0.0125	0.6090 ± 0.0121	0.5284 ± 0.0539	0.6751 ± 0.0048	0.8705 ± 0.0004	0.7902 ± 0.0013
GraphSAGE	0.6363 ± 0.0141	0.8353 ± 0.0024	0.7550 ± 0.0027	0.7777 ± 0.0176	0.9445 ± 0.0033	0.8894 ± 0.0070
GPRGNN	0.6175 ± 0.0107	0.7439 ± 0.0023	0.6342 ± 0.0456	0.8335 ± 0.0954	0.9282 ± 0.0193	0.8597 ± 0.1240
CARE-GNN	0.6104 ± 0.0033	0.7710 ± 0.0009	0.7048 ± 0.0006	0.8921 ± 0.0007	0.9401 ± 0.0056	0.8852 ± 0.0002
PC-GNN	0.6412 ± 0.0000	0.7782 ± 0.0000	0.7134 ± 0.0000	0.8817 ± 0.0000	0.9665 ± 0.0000	0.8950 ± 0.0000
CONSISGAD	0.6242 ± 0.0540	0.8323 ± 0.0083	0.4709 ± 0.1133	0.8854 ± 0.0148	0.9320 ± 0.0041	0.8560 ± 0.0113
SpaceGNN	0.5717 ± 0.0004	0.6561 ± 0.0013	0.4462 ± 0.0041	0.8924 ± 0.0024	0.9308 ± 0.0028	0.8456 ± 0.0081
GHRN	0.6794 ± 0.0037	0.8110 ± 0.0027	0.6774 ± 0.0208	0.9026 ± 0.0084	0.9667 ± 0.0036	0.8913 ± 0.0042
BWGNN	0.7071 ± 0.0107	0.8378 ± 0.0088	0.6898 ± 0.0254	0.9126 ± 0.0030	0.9624 ± 0.0010	0.8953 ± 0.0096
TROPICAL	0.7543 ± 0.0068	0.8906 ± 0.0035	0.7894 ± 0.0090	**0.9142** **±** **0.0054**	0.9345 ± 0.0137	0.8923 ± 0.0065
HCLNet	**0.7701** **±** 0.0066^*^	**0.9105** **±** 0.0010^*^	**0.8212** **±** 0.0033^*^	0.8944 ± 0.0043	**0.9702** **±** 0.0018^*^	**0.8968** **±** 0.0033^*^

For each metric, the top-1 and top-2 performers among all models are highlighted in boldface and underlined, respectively. An asterisk (^*^) indicates that HCLNet's performance improvement over the best baseline is statistically significant by the t-test (*p* < 0.01).

### 4.3 Ablation study

To systematically evaluate the effectiveness of each innovative component in HCLNet, we design two sets of ablative experiments. First, to validate the overall contribution of combining MG-HAM and HDV-CL in HCLNet, we construct two fundamental variants: **(a)** HCLNet-hg, which removes the hypergraph module and employs an MLP for node feature updating, while retaining HDV-CL's feature augmentation and node-level contrastive learning, and **(b)** HCLNet-cl, which completely removes the HDV-CL module. By comparing the fraud detection performance of these variants against the full HCLNet, we assess the individual contributions of each component to the overall model performance.

**Figure 2:**
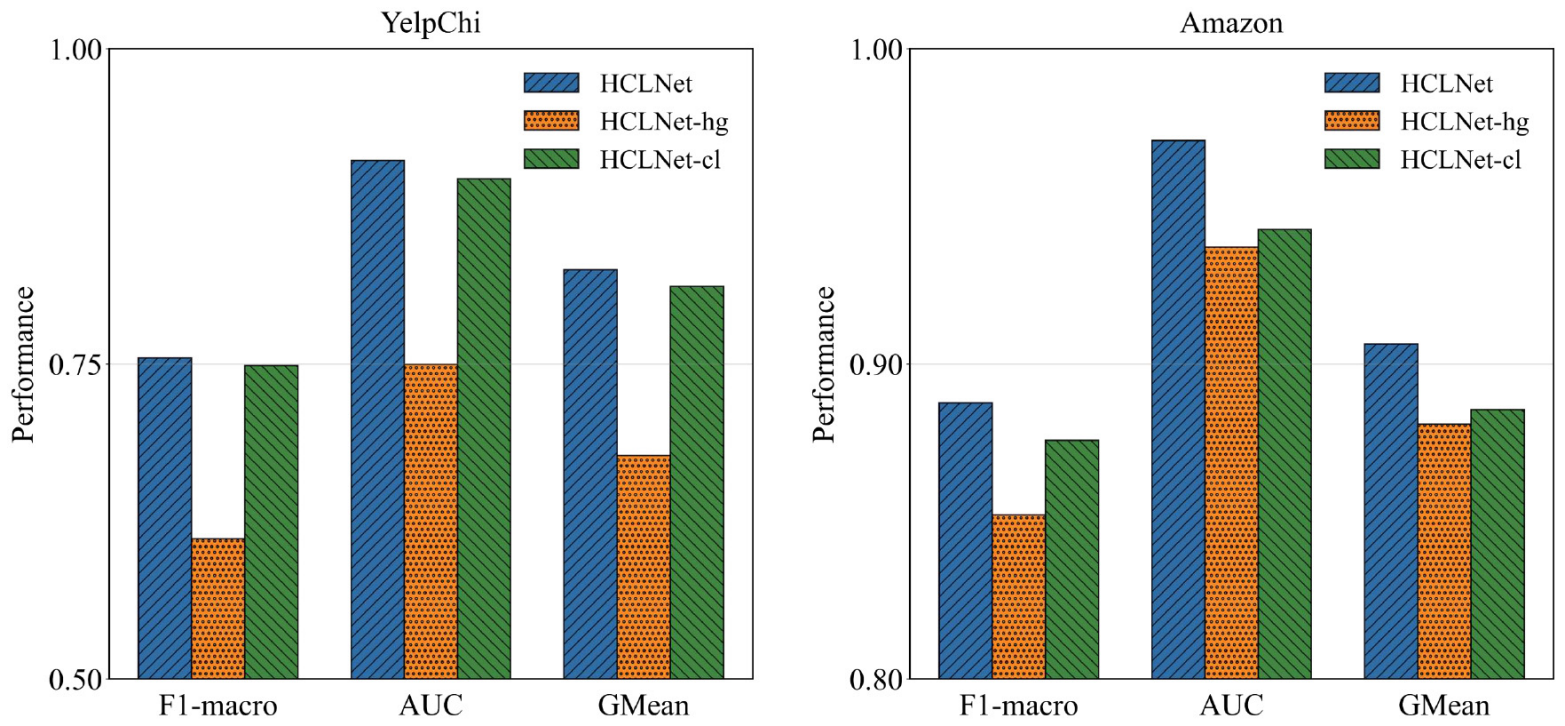
Ablation study on two key components (mean over 10 runs). Statistical significance of pairwise differences of HCLNet against the best variant is determined by the *t-*test (*p* < 0.01).

As shown in Figure 2, on both datasets, HCLNet achieves optimal performance across all three evaluation metrics. Removing the hypergraph module and MG-HAM in HCLNet significantly degrades performance, underscoring the critical role of the hypergraph structure in capturing complex fraud patterns. Conversely, while retaining the hypergraph module, removing HDV-CL in HCLNet still results in inferior performance compared to the complete HCLNet, confirming the essential contribution of contrastive learning to feature discriminability. Notably, HCLNet-cl outperforms HCLNet-hg, suggesting that HDV-CL offers stronger robustness even in the absence of the hypergraph structure. Collectively, the synergy between MG-HAM and HDV-CL enables HCLNet to effectively model complex relational patterns and feature invariance for fraud detection.

On the basis of confirming the effectiveness of the overall framework, we further deconstruct the HDV-CL by constructing two additional variants: **(b_1_)** HCLNet-nd, which removes node-level contrastive learning, and **(b_2_)** HCLNet-hp, which removes hyperedge-level contrastive learning, to investigate their individual contributions. The experimental results shown in Table 3 indicate that removing either level of contrastive learning leads to performance degradation, confirming the necessity of the hierarchical design. It is worth noting that the absence of node-level contrastive learning has a more significant impact on the model, indicating that learning discriminative node features is fundamental for improving detection performance. In contrast, hyperedge-level contrastive learning provides valuable supplementary information by capturing group-wise semantic consistency. Together, they form a hierarchical feature learning framework that enhances the model's ability to identify fraud patterns from both individual and group perspectives.

**Table 3:** Ablation study on HDV-CL components (mean ± std over 10 runs).

**Variants**	**YelpChi**			**Amazon**		
	**F1-macro**	**AUC**	**GMean**	**F1-macro**	**AUC**	**GMean**
HCLNet-nd	0.7484 ± 0.0033	0.8895 ± 0.0011	0.8046 ± 0.0059	0.8863 ± 0.0058	0.9609 ± 0.0069	0.8906 ± 0.0086
HCLNet-hp	0.7601 ± 0.0107	0.9099 ± 0.0033	0.8205 ± 0.0014	0.8858 ± 0.0099	0.9673 ± 0.0029	0.8916 ± 0.0061
HCLNet-cl	0.7503 ± 0.0040	0.8947 ± 0.0017	0.8054 ± 0.0080	0.8801 ± 0.0065	0.9498 ± 0.0068	0.8844 ± 0.0076
HCLNet	**0.7701** **±** 0.0066^*^	**0.9105** **±** 0.0010^*^	**0.8212** **±** 0.0033^*^	**0.8944** **±** 0.0043^*^	**0.9702** **±** 0.0018^*^	**0.8968** **±** 0.0033^*^

An asterisk (^*^) indicates that HCLNet's performance improvement over the best baseline is statistically significant by the *t*-test (*p* < 0.01). The highest scores are shown in bold.

In summary, the organic integration of MG-HAM and HDV-CL enables HCLNet to simultaneously leverage the structural representation capabilities of hypergraphs and the feature enhancement advantages of contrastive learning. Furthermore, the node-level and hyperedge-level components within the HDV-CL collectively enhance the model's overall performance in fraud detection tasks through feature optimization at different granularities.

### 4.4 Parameter sensitivity

**Figure 3:**
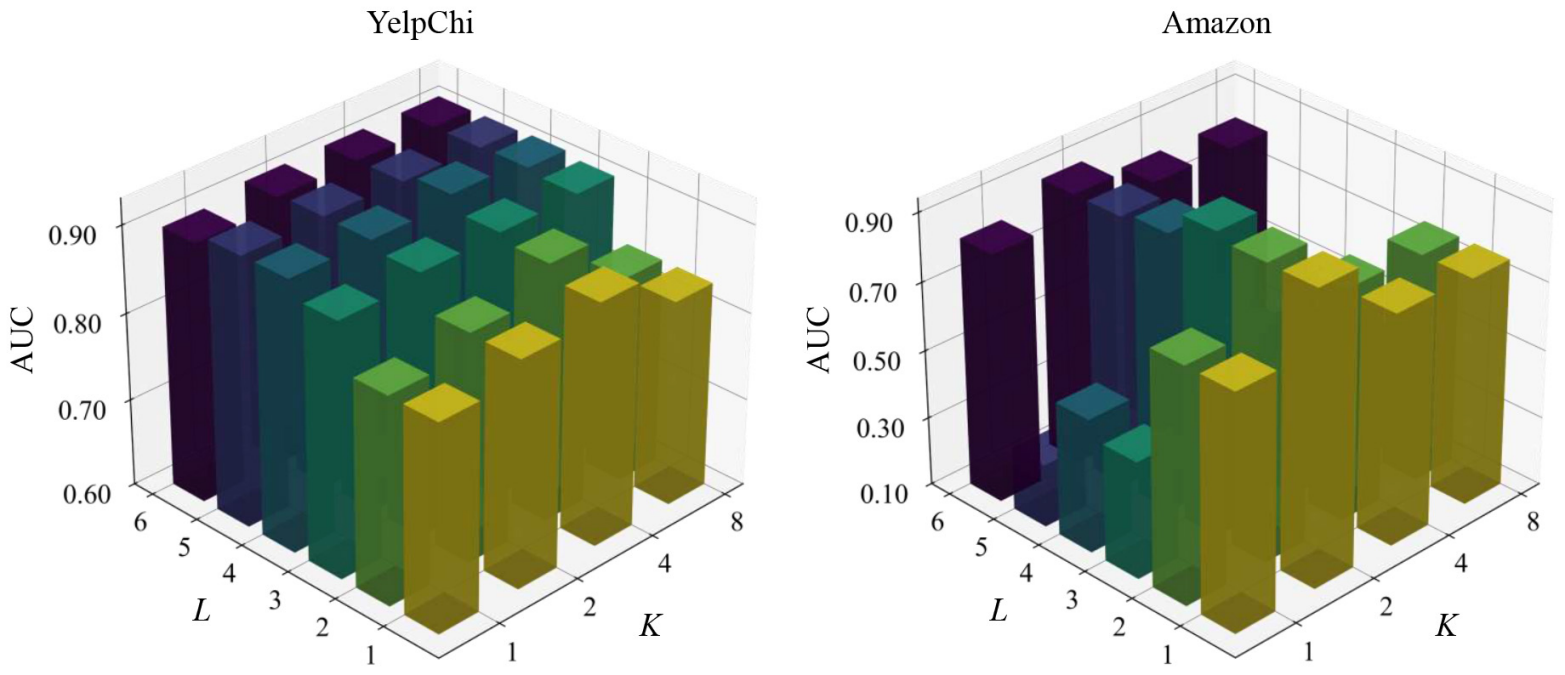
Parameter sensitivity of *L* and *K* on YelpChi and Amazon datasets. Note that the values of *L* range from [1, 2, 3, 4, 5, 6] and *K* range from [1, 2, 4, 8].

The proposed HCLNet incorporates two critical hyperparameters, i.e., layer depth *L* governing hypergraph convolution and hierarchical dual-view contrastive learning, and head number *K* controlling multi-head hypergraph convolution. To evaluate HCLNet's robustness across hyperparameter configurations, we conducted parameter sensitivity analysis on YelpChi and Amazon datasets, systematically varying *L* ∈ {1, 2, 3, 4, 5, 6} and *K* ∈ {1, 2, 4, 8} to examine their impact on fraud detection performance. Figure 3 presents the parameter sensitivity results.

Experimental results demonstrate distinct patterns across datasets. YelpChi exhibits robust depth scalability, where performance progressively improves as layers increase beyond the third, stabilizing between the fourth and fifth layer. This optimization stems from multi-head attention synergy. Under four-head configuration, hierarchical contrastive learning effectively coordinates feature representations across relational perspectives while preserving task-specific focus. This collaborative mechanism reaches optimal balance at the fourth layer, with gated residual connections mitigating performance degradation even at the sixth layer.

Conversely, Amazon peaks at the third layer with dual-head configuration, attributable to specialized division of labor: one head processes dominant user browsing relationships while the other isolates critical co-purchasing signals. This inter-head isolation enables precise capture of decisive fraud evidence at optimal depth. However, further layer increases degrade intermediate-layer performance (4th–5th layer), where superfluous heads introduce noise and additional layers dilute critical signals through dominant relationships. Significantly, all head configurations recover substantially at the sixth layer through gated residual connections. Upon reaching critical depth, the gating mechanism automatically fuses third-layer key signals with shallow features, bypassing intermediate contamination through cross-layer feature recombination.

In conclusion, parameter sensitivity analysis reveals fundamental configuration principles: YelpChi benefits from progressive multi-head collaboration across diverse relationships, while Amazon requires precise inter-head isolation and depth-specific configuration. This divergence reflects inherent topological differences in fraud patterns—concentrated multidimensional signals vs. fragile critical evidence requiring protection. The universal sixth-layer recovery on Amazon ultimately demonstrates that in skewed relational networks, effective depth design prioritizes cross-layer feature preservation and recombination pathways over unlimited feature transformation.

### 4.5 Interpretability exploration

We conducted a visual analysis of node embeddings on the YelpChi graph dataset. To intuitively compare the performance of different models, we employed t-SNE technique (van der Maaten, 2008) to map the outputs from the classification layer of various models, just before their final layers, into a two-dimensional space for dimensionality reduction. This visualization technique enables clear observation and analysis of differences in the outputs of the models.

**Figure 4:**
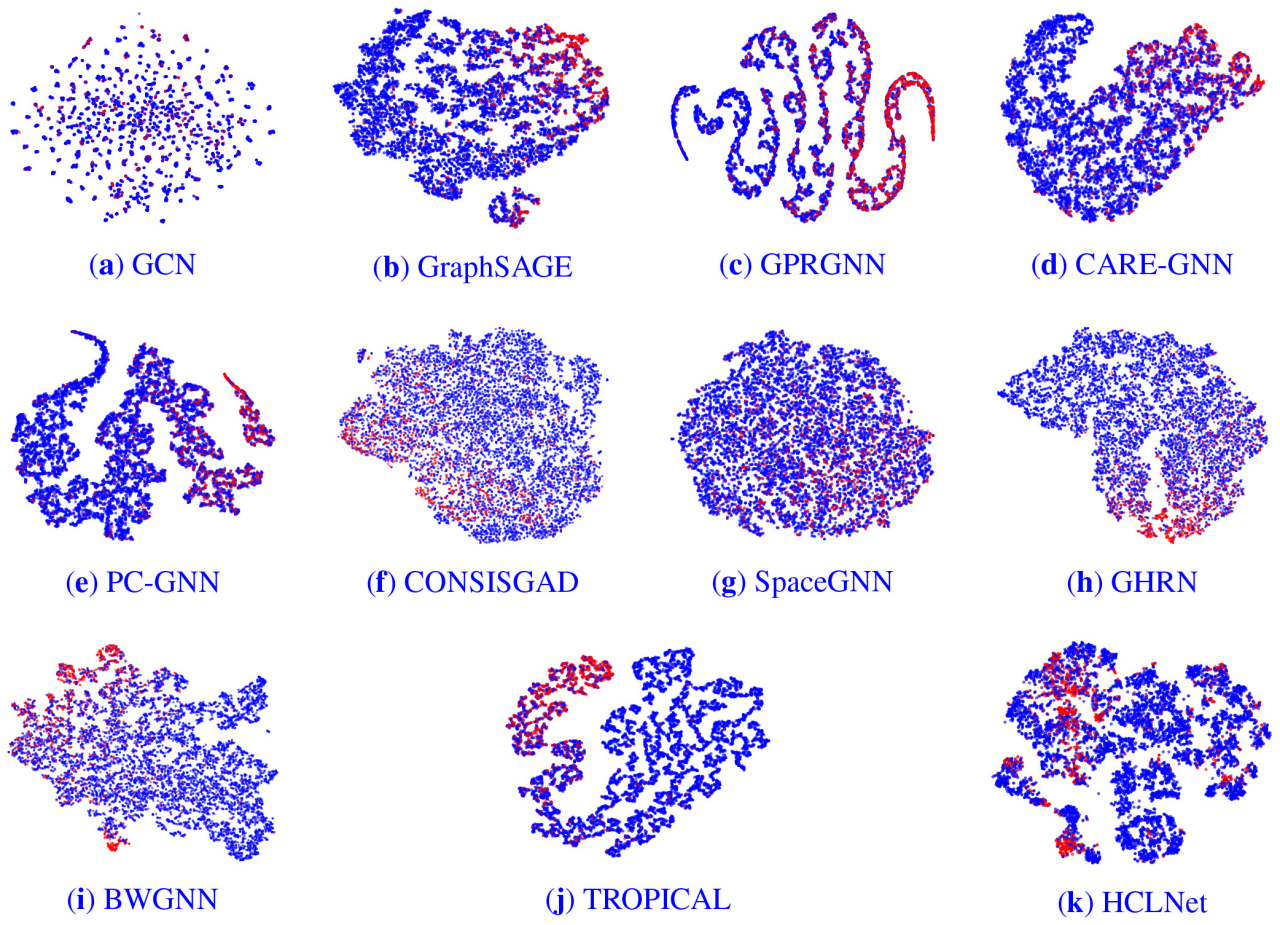
Embedding visualization of different models. The red and blue nodes represent fraudsters and benign entities respectively. **(a)** GCN. **(b)** GraphSAGE. **(c)** GPRGNN. **(d)** CARE-GNN. **(e)** PC-GNN. **(f)** CONSISGAD. **(g)** SpaceGNN. **(h)** GHRN. **(i)** BWGNN. **(j)** TROPICAL. **(k)** HCLNet.

The results, displayed in Figure 4, show fraud nodes in red and benign nodes in blue. Figures 4a–e correspond sequentially to the baseline models listed in Table 2, while f presents the visualization result for HCLNet. In contrast to the other models, HCLNet exhibits a distinct “isolation belt” effect. Within its visualization, fraudulent and benign nodes form highly separated clusters with sharp boundaries, clearly demarcated by a low-density transition zone between them. This structural characteristic stems directly from the synergistic interplay of HCLNet's MG-HAM and HDV-CL. Specifically, MG-HAM captures diverse relational patterns in the hypergraph through its multi-head attention mechanism operating in parallel. Each attention head focuses on feature interactions within distinct semantic subspaces. Concurrently, gated residual connections dynamically regulate the weighting between previous and newly generated features. This ensures crucial discriminative information is enhanced and preserved while suppressing noise. This design fosters the formation of node embeddings that exhibit high intra-group cohesion and low inter-group coupling within the feature space.

Furthermore, HDV-CL explicitly enhances inter-class separability through its dual-view node-level and hyperedge-level contrastive learning optimization. At the node level, it forces embeddings of nodes belonging to the same class to converge while pushing apart embeddings of nodes from different classes. At the hyperedge level, it learns globally discriminative structures by contrasting representations of hyperedges with different semantics. Dual-view data augmentation further mimics real-world noise interference, compelling the model to uncover essential features rather than superficial correlations. In contrast, other models lack such structured representation learning mechanisms and fail to achieve comparable separation efficacy, resulting in poorer performance on the fraud detection task.

## 5 Conclusion and future work

In this paper, we propose a novel hypergraph contrastive learning framework called HCLNet for fraud detection that addresses three core challenges: homophily assumption failure, extreme label imbalance, and inadequate high-order modeling. Through multi-relational hypergraph fusion, we encode complex fraud syndicates into hyperedges to explicitly capture collaborative fraud patterns. The multi-head gated hypergraph aggregation mechanism (MG-HAM) dynamically filters noise while preserving discriminative signals via parallel semantic subspace learning and gated residual connections. Complementarily, our hierarchical dual-view contrastive learning framework (HDV-CL) harnesses self-supervision at both node and hyperedge levels to enhance feature separability under label scarcity.

Despite promising results, our work still has several limitations that warrant further investigation in future work. First, evaluation on only two public datasets (YelpChi and Amazon) limits generalizability. Future work should validate HCLNet in broader fraud scenarios like financial transactions and insurance fraud to assess cross-domain adaptability. Second, hypergraphs may face scalability challenges with large-scale dynamic graphs due to computational overhead. Future research should focus on optimizations, such as sampling-based or hierarchical hypergraph processing strategies, to improve computational efficiency. Third, inherent biases in public datasets, such as platform-specific user behaviors and annotation inconsistencies, may impact model performance and fairness. Therefore, future studies should incorporate more diverse and representative data sources and explore the integration of debiasing techniques during training. We believe HCLNet's core ideas offer a valuable framework for fraud detection, though realizing its full potential may require domain-specific adaptations.

## Data Availability

Publicly available datasets were analyzed in this study. This data can be found here: The YelpChi and Amazon graph datasets used in this study are publicly available. The YelpChi dataset can be accessed at https://www.yelp.com/dataset. The Amazon dataset is available through the Amazon Product Review API or at https://nijianmo.github.io/amazon/index.html.
